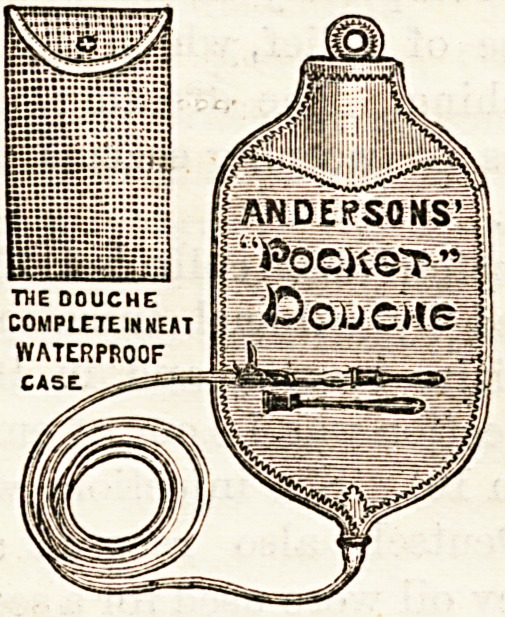# New Appliances and Things Medical

**Published:** 1899-09-02

**Authors:** 


### NEW APPLIANCES AND THINGS MEDICAL.

[We shall be glad to receive, at our Office, 28 & 29, Southampton Street, Strand, London, W.C., from the manufacturers, specimens of all new preparations and appliances which may be brought out from time to time.]

### NEW POCKET DOUCHE.

(Anderson, Anderson, and Anderson, 37, Queen Victoria Street, London, E.C.)

This douche is made of indiarubber in the usual bag form, and is supplied in various sizes, from one to four quarts. As sent out complete, it consists of bag, six feet of tubing, with vaginal and rectal tubes. The whole is contained in a small waterproof case like an elongated sponge bag. Being small in compass, it is easily carried in the pocket or bag. For district nurses this form of douche should prove of great convenience, and in midwifery practice it has obvious advantages. As compared with the old familiar rubber syringe the douche is infinitely superior, and capable of more exact adjustment as regards the pressure of water employed.

### FRIARS' MEDICATED AND OTHER SOAPS.

(Friars' Soap Company, 1, Valentine Place, Blackfriars, London, S.E.)

Friars' medicated soap consists of a basis of soap, which is thoroughly sound in itself, evidently of careful manufacture, and made from fat of good quality. It is impregnated with an antiseptic of the eucalyptus variety. The odour is distinctly agreeable, and the soap is such that it will be appreciated as an ordinary toilet adjunct by the most fastidious, and possessing, as it does, excellent antiseptic qualities, it is particularly indicated in cases where there is any likelihood of infection, as in hospitals, nursing homes, and institutions, or in private houses during times of epidemic. Friars' cream soap, which is intended for toilet use, and possesses no particular medicinal ingredients, is a pleasant emollient soap of good quality, exceedingly fragrant. It is practically neutral in reaction, and hence can contain no free caustic soda. It contains a low percentage of moisture, and is consequently an economical soap, and being free from injurious colouring matter and other poisons is particularly to be recommended for nursery use and for application to delicate skins. A very excellent shaving soap is supplied by the same firm in convenient tins. This soap rapidly works up into a good lather, and remains in that condition longer than most soaps we have tried. It is thus a great convenience to those who have to shave daily. It leaves the skin soft, and appears to promote a healthy condition of the integument.

### SUCHARD'S CHOCOLATE.

We have received samples of the above chocolate, and find that both the edible and the cooking varieties are of the purest description. The delicacy of flavour is, perhaps, the most distinguishing feature, for at the present day, owing to public demands, many other brands are quite pure, but in many instances there is a distinct crudeness about the flavour. Suchard's, on the other hand, is refined and delicate in flavour. For making a cup of chocolate we highly recommend this brand. If not made too strong and with no added sugar the beverage is refreshing and nutritious, without being thick and sickly, as is the case when one orders a cup of chocolate at a restaurant and cheaper brands are employed. Chocolate Suchard can now be obtained ready mixed with condensed milk. By mixing the paste with hot water a most delicious cup of chocolate can be obtained at no trouble and little expense. The ingredients are of the purest, the milk carefully and thoroughly sterilised, and from a medical and hygienic point of view greatly to be desired for invalids and children.

## Figures and Tables

**Figure f1:**